# Southeast Texas Medical Society

**Published:** 1893-09

**Authors:** 


**The Southeast Texas Medical Society** met in regular session in Beaumont, Tex., on the 25th day of July, 1893. Vice-President Thompson took the chair, and J. Saunders was appointed Secretary pro tem. The Chairman announced that the election of officers would take place. Election resulted as follows: President, A. N. Perkins, of Sabine Pass; First Vice-President, C. Y. Thompson, of Beaumont; Second Vice-President, T. B. Sellman, of Village Mills; Secretary and Treasurer, J. Saunders, of Orange. The Board of Censors to be appointed by President Perkins.

Dr. Price reported a case of villous growth with hypertrophy of the bladder, treated by suprapubic section and application of pyoktanin blue.

Dr. Sholars reported a case of obstructed bowels in a saw filer, whose alimentary canal contained large quantities of emery dust.

Dr. Thompson reported a case of compound fracture of skull involving duramater, treated by open wound dressing, with recovery.

On motion, the by-law making Beaumont the permanent place of meeting be changed at the next regular meeting so that it may include other places in southeast Texas.

Motion was made and caıried that a general invitation be extended to the regular physicians of all southeast Texas, and each member of the society be requested to write to physicians and urge them to be present at our next meeting and become members.

After an interesting discussion of the cases reported, it was, on motion, ordered that the subject of bowel diseases of children be discussed at the next meeting of this Society, which will take place in Beaumont on the second Tuesday in October next, and that Dr. Seastrunk open the discussion.

